# A multi-analyte liquid biopsy approach for nonseminomatous testicular germ cell tumors: combining cfDNA and N-glycan analysis in blood and seminal plasma

**DOI:** 10.1186/s12935-025-03887-8

**Published:** 2025-07-11

**Authors:** Jure Krasic, Dinko Soic, Lucija Skara Abramovic, Ivona Kolosnjaj, Miroslav Tomic, Alen Vrtaric, Sasa Kralik Oguic, Nina Gelo, Ana Katusic Bojanac, Davor Jezek, Dinko Mitrecic, Monika Ulamec, Tomislav Kulis, Olga Gornik, Nino Sincic

**Affiliations:** 1 Department of Histology and Embryology, School of Medicine, University of Zagreb, Šalata 3, 10000 Zagreb, Croatia; 2 Laboratory for Stem Cells, Croatian Institute for Brain Research, School of Medicine, University of Zagreb, 10000 Zagreb, Croatia; 3 Centre of Excellence for Reproductive and Regenerative Medicine, School of Medicine, University of Zagreb, 10000 Zagreb, Croatia; 4 Faculty of Pharmacy and Biochemistry, University of Zagreb, 10000 Zagreb, Croatia; 5 Department of Virology, Croatian Institute of Public Health, 10000 Zagreb, Croatia; 6 Department of Urology, University Clinical Hospital Centre Sestre Milosrdnice, 10000 Zagreb, Croatia; 7 Department of Clinical Chemistry, University Clinical Hospital Center Sestre Milosrdnice, 10000 Zagreb, Croatia; 8 Clinical Institute of Laboratory Diagnostics, University Hospital Centre Zagreb, 10000 Zagreb, Croatia; 9 Department of Clinical Embryology, University Hospital Centre Zagreb, 10000 Zagreb, Croatia; 10 Department of Biology, University of Zagreb School of Medicine, 10000 Zagreb, Croatia; 11 BIMIS — Biomedical Research Center Šalata, School of Medicine, University of Zagreb, 10000 Zagreb, Croatia; 12 Ljudevit Jurak Clinical Department of Pathology and Cytology, University Clinical Hospital Center Sestre Milosrdnice, 10000 Zagreb, Croatia; 13 Department of Pathology, School of Medicine, University of Zagreb, 10000 Zagreb, Croatia; 14 Department of Urology, University Hospital Centre Zagreb, 10000 Zagreb, Croatia; 15 Department of Urology, School of Medicine, University of Zagreb, 10000 Zagreb, Croatia

**Keywords:** Cell-free DNA, N-glycosylation, Epigenetics, Biomarkers, Testicular germ cell tumors, Semen quality

## Abstract

**Background:**

Nonseminomatous testicular germ cell tumors (NSE) present significant diagnostic challenges, especially for the early detection of serum tumor marker (STM) negative cases. Current diagnostic tools are limited, highlighting the need for innovative approaches. This study investigates a novel multi-analyte approach combining circulating cell-free DNA (cfDNA) and N-glycan profiling in both blood and seminal plasma to improve NSE diagnostics.

**Methods:**

The study included 41 NSE patients and 114 healthy controls. Diagnostic potential of cfDNA parameters (quality and fragmentation), cfDNA methylation (*RASSF1A*, *PRSS21*, and *LINE-1*) and N-glycan alterations in blood plasma and seminal plasma samples was investigated using logistic regression models. Pre- and post-radical orchidectomy longitudinal samples from NSE patients were analyzed to assess surgical treatment response and disease monitoring utility.

**Results:**

Blood plasma analysis of combined cfDNA and N-glycan profiling demonstrated high diagnostic precision, with an AUC of 0.96, identifying 85% of STM-negative patients and all pure-form teratomas. Post-operative blood plasma analysis showed that *LINE-1* cfDNA methylation levels returned to those of healthy controls. Seminal plasma analysis revealed an increased cfDNA fragmentation index (CFI) and cfDNA methylation changes in *LINE-1* and *PRSS21*, with an AUC of 0.83 and identifying 85% of STM-negative patients.

**Conclusion:**

The proposed multi-analyte approach significantly improves early diagnostics of NSE, particularly for STM-negative cases and teratomas. *LINE-1* cfDNA methylation is a promising biomarker for NSE detection and treatment monitoring. These findings could transform diagnostic strategies and patient management in testicular germ cell tumors, with potential applications in reducing overtreatment and improving outcomes .

**Graphical Abstract:**

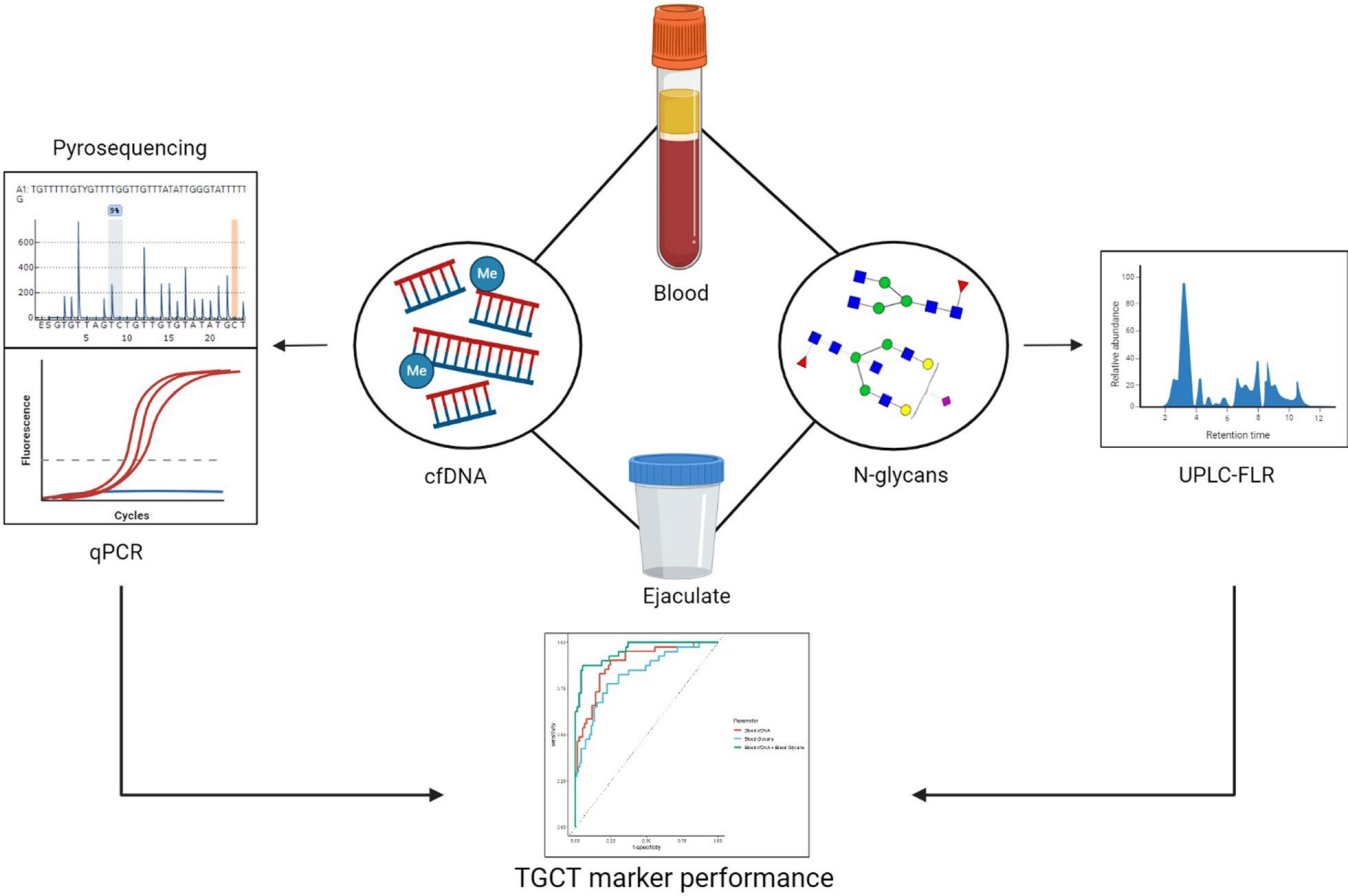

**Supplementary Information:**

The online version contains supplementary material available at 10.1186/s12935-025-03887-8.

## Introduction

Testicular germ cell tumors (TGCTs) are the most common neoplasm in 15–44 year old males [1] with a rising global incidence of over 70% in the last 20 years [2, 3]. TGCTs are a highly complex group of tumors with heterogenous histology further subdivided into pure seminomas (SE) and nonseminomas (NSE), both of which occur about equally [4, 5]. However, prognosis is worse in patients with NSE, with disease recurrence occurring in 20–40% of patients [6]. NSE subtypes vary histologically and molecularly and include embryonal carcinomas (EC), yolk sac tumors (YST) along with potentially chemo-resistant teratomas (TE) and choriocarcinomas (CH) [4, 7]. Two or more TGCT subtypes are often present in the same tumor, then classified as mixed testicular germ cell tumors (MTGCTs). MTGCTs are one of the most histologically complex tumors [8, 9] and have worse prognosis than pure form EC [10].

The current protocol for diagnosis of TGCT is the pathohistological examination of the testis excised by radical orchidectomy. Tissue biopsies are not performed in cases of suspect TGCT due to very high chance of tumor seeding [11]. Due to this there has been an increase in interest in liquid biopsies, which offer a minimally invasive way to approach hard-to-reach or dangerous-to-sample tumors [12]. Current clinical serum tumor markers (STM), such as α-fetoprotein (AFP), β-human chorionic gonadotropin (bHCG), and lactate dehydrogenase (LDH), have limited diagnostic utility due to their low sensitivity and specificity [11, 13]. The detection of STM negative tumors remains an unanswered need in TGCT patient management [14].

Investigation of “first line” cell-free DNA (cfDNA) diagnostic biomarkers (namely cfDNA quality, fragmentation, and methylation) can discriminate healthy individuals from cancer patients, is easily performed and offers comprehensive disease insight [12, 15, 16]. For cfDNA methylation analyses we have selected two tumor suppressor genes known to be inactivated by DNA promoter hypermethylation in TGCT and especially NSE (*RASSF1A* and *PRSS21*) and *LINE-1* as surrogate marker for global DNA methylation, due to 500000 copies of *LINE-1* being found per genome [17–26]. While genetic analyses of cfDNA are common in cancer research, TGCTs are characterized by a low mutational burden and the absence of alterations in driver genes, pointing to the main role being epigenetic regulations in TGCT pathogenesis [27]. In particular, levels of DNA methylation differ greatly between TGCT histological subtypes indicating its potential in diagnostic application [28, 29]. N-linked glycosylation is an impactful co- and post-translational modification of proteins increasingly researched because of its diagnostic, monitoring and prognostic potential in various diseases [30–32]. However, despite currently STM’s AFP and bHCG both being glycoproteins, N-glycosylation investigations of TGCTs have been scarce, with only one study investigating serum N‐glycan profiling [33, 34].

This study presents a multianalyte approach combining cfDNA and N-glycan profiling in blood and seminal plasma to address the critical diagnostic challenges in NSE. NSEs are characterized by greater histological and molecular complexity and a worse prognosis compared to seminomas, yet current diagnostic tools, particularly for serum tumor marker (STM)-negative cases and TE patients, remain inadequate. The reliance on STMs for early diagnosis is limited by their low sensitivity and specificity [13], while possible future clinical markers such as miR-371a-3p which should solve STM-negative patient detection are unable to detect TE [35]. To bridge this gap, we analyzed samples from NSE patients before and after radical orchidectomy, alongside healthy controls. We investigated novel diagnostic biomarkers, including cfDNA parameters (concentration, integrity, and methylation of *RASSF1A*, *PRSS21*, and *LINE-1*) and N-glycan profiles in blood and seminal plasma. To our knowledge, this is the first study to integrate cfDNA and N-glycomic analyses in NSE, offering a comprehensive and minimally invasive approach to improve diagnostic accuracy. Our findings demonstrate the potential of this multi-analyte strategy to enhance the detection of STM-negative NSE cases and provide valuable insights into treatment monitoring through the complementary analysis of circulating biomarkers in both blood and seminal fluid.

## Materials and methods

### Patients

Prospective NSE patients were recruited from the urology departments of the University Hospital Centre Zagreb and the University Hospital Centre Sestre Milosrdnice after the confirmation of the NSE diagnosis by a pathologist (following radical orchidectomy) while still treatment naive. In total 41 patients were included in the study. STM’s were analyzed pre-orchidectomy (AFP, LDH and bHCG) and were classified as either STM positive (elevation of any one marker) or negative (no STM elevated). A total of 114 healthy controls were recruited from the general male population, with no prior diagnosis of TGCT, of self-reported good health and were age-matched to patients (18–46 years).

### Ethical statement

Information about the study was given to all the participants who gave written consent. The study was conducted according to the Declaration of Helsinki. The Ethics Committee of the School of Medicine University of Zagreb (protocol code 380-59-10106-23-111/37, 641-01/23-02/01), University Hospital Centre Sestre Milosrdnice (protocol code, number: EP-18327/17-3) and University Hospital Centre Zagreb (protocol code 8.1-18/72-2, number: 02/21 AG) approved the collection and manipulation of all samples.

### Sample collection and preparation

Blood and ejaculate were obtained from NSE patients and healthy controls. Liquid biopsy samples from NSE patients were collected pre- and post-radical orchidectomy. Pre-operative samples were obtained up to 2 days before radical orchidectomy, and post-operative samples were obtained 8 days after radical orchidectomy (before any chemo or radiotherapy).

### Plasma preparation was performed as described previously [36]

In short, 12 mL of peripheral venous blood were collected into two 6 mL EDTA containing tubes (Greiner Bio-One, Kremsmünster, Austria). The blood was further processed within 2 h of venipuncture. Blood plasma was obtained by double-centrifugation (1400×g for 10 min and 4500×g for 10 min) and was stored at -80 °C.

Ejaculate samples were obtained parallel to blood samples and were obtained after 3–5 days of sexual abstinence. Ejaculate samples were allowed to liquefy for 30–60 min at RT before further processing. Seminal plasma was obtained by triple centrifugation (400×g for 10 min, 12,000×g for 10 min, and 20,000×g for 10 min) and was stored at −80 °C.

### cfDNA isolation and quantification

cfDNA was isolated according to a modified protocol using the NucleoSnap cfDNA kit (Machery-Nagel, Düren, Germany) and the QIAvac 24 Plus vacuum station (QIAGEN, Hilden, Germany) as previously described [36]. cfDNA was eluted in 100 µL of elution buffer and stored at −80 °C.

Quantification of isolated cfDNA was done by qPCR on the CFX96 Touch Real-Time PCR Detection System (Bio-Rad Laboratories, Hercules, CA, USA) using SsoAdvanced Universal SYBR Green Supermix (Bio-Rad Laboratories, Hercules, CA, USA) and *LINE-1* fragment primers, as previously described [36]. Two *LINE-1* fragments were quantified, a short 82 bp and a long 224 bp fragment were quantified with the ratio of long-to-short fragments being used to calculate the cell-free DNA integrity score (CFI) [37].

### cfDNA methylation analysis

Bisulfite conversion of cfDNA was performed using the EpiTect Plus DNA Bisulfite Kit (QIAGEN, Hilden, Germany) according to the manufacturer’s instructions. Bisulfite-converted cfDNA was eluted in 20 µL of elution buffer and stored at −80 °C, as described previously [36].

The PCR and pyrosequencing primers were designed using PyroMark Assay Design 2.0 (QIAGEN, Hilden, Germany) and are shown in Supplementary Table 1. Bisulfite-converted cfDNA was PCR amplified according to the previously published protocols [36]. The biotinylated PCR product was purified using the Pyromark Q24 Vacuum Workstation (Qiagen, Hilden, Germany). Pyrosequencing was performed by the Pyromark Q24 Advanced System with PyroMark Q24 CpG Advanced Reagents (Qiagen, Hilden, Germany). cfDNA methylation levels were calculated as the ratio of C/T at a CpG site using the Pyromark Q24 Advanced Software 3.0.1 (Qiagen, Hilden, Germany). Average cfDNA methylation was calculated as the average value of the investigated CpG’s per gene of interest [36].

### Enzymatic release and fluorescent labeling of N-Glycans from plasma proteins

A high-throughput N-glycosylation analysis of blood plasma proteins was performed using the HILIC-UHPLC method, described previously in detail [38], with small modification for the N-glycan analysis of seminal plasma proteins. Prior to analysis, plasma samples were randomly distributed on 96-well sample collection plates. For N-glycan analysis of both blood and seminal plasma proteins a 10 μL aliquot of plasma was used. Plasma samples were denatured by adding SDS (Invitrogen, Carlsbad, CA, USA) and incubated at 65 °C for 10 min. After denaturation, Igepal-CA630 (Sigma-Aldrich, St. Louis, MO, USA) was added and shaken for 15 min at room temperature.

Enzymatic release of N-glycans was achieved by addition PNGaseF (Promega, Madison, WI, USA) and overnight incubation at 37 °C. The released glycans of blood plasma proteins were fluorescently labeled with 2-aminobenzamide (2-AB), while for seminal plasma glycans procainamide was used. The freshly prepared labeling mixture consisted of 2-AB or procainamide (Sigma-Aldrich, St. Louis, MO, USA), 2-picoline borane (Sigma-Aldrich, St. Louis, MO, USA) and acetic acid/water (30:70, vol/vol) (Merck, Darmstadt, Germany) in dimethyl sulfoxide (DMSO) (Sigma-Aldrich, St. Louis, MO, USA). The labeling reaction was carried out at 65 °C for 2 h. The labeled glycans were isolated from the reaction mixture by solid phase extraction (SPE) to remove residues of the labeling solution, reducing substances and other impurities. After labeling, N-glycan clean-up was performed using 0.2 μm SUPOR filter plate (Pall Corporation, New York, NY, USA). The labeled N-glycans were eluted from the plates with ultra-pure water and stored at −20 °C until subjected to chromatography analysis.

### Hydrophilic interaction liquid chromatography of labeled N-glycans

The fluorescently labeled N-glycans were separated by hydrophilic interaction chromatography (HILIC) on an Acquity UPLC H-class instrument (Waters, Milford, MA, USA). The excitation and emission wavelengths for 2-AB were set to 250 nm and 428 nm, that is 310 nm and 370 nm for procainamide respectively. The plasma N-glycans were separated on a 150 × 2.1 mm Glycan BEH Amide column (Waters, Milford, MA, USA). Solvent A consisted of a 100 mmol/L solution of ammonium formate in water with a pH of 4.4, while solvent B consisted of 100% ACN (LC–MS purity). A linear elution gradient of 30–47% of solvent A at a flow rate of 0.561 mL/min in a 25-min analytical run was used for the analysis of blood plasma proteins N-glycans, while for seminal plasma N-glycans a gradient of 32–47% was used. The separation temperature was maintained at 25 °C and samples were cooled to 10 °C prior to injection.

Data were processed using an automated processing method, after which each chromatogram was manually corrected to ensure uniform integration intervals between samples. The chromatograms were separated into 39 peaks (GP1-GP39) for blood plasma N-glycans and 31 peaks (SGP1-SGP39) for seminal N-glycans respectively. N-glycan values were expressed as a percentage of total integrated area, while the glycan composition of each blood plasma N-glycan peak had been previously confirmed in studies using LC–MS [39].

### Sample processing success rates

All samples successfully produced results for cfDNA assays, while one control blood-plasma sample, one patient seminal-plasma sample and one patient blood-plasma sample yielded insufficient volume during HILIC-UHPLC preparation and were excluded from N-glycan analysis.

### Statistical analysis

Methylation levels were transformed into M scores before further processing. For normalization purposes cfDNA amounts and CFI were log2 transformed. Normality was tested using Shapiro–Wilk test. The differences in M scores, cfDNA amounts and CFI between preoperative samples and healthy controls were assessed using the Wilcoxon rank-sum (Mann–Whitney U) test with Benjamini–Hochberg correction for multiple testing. The differences in M scores, cfDNA amounts and CFI between preoperative samples postoperative samples were assessed using the Wilcoxon signed-rank test with Benjamini–Hochberg correction for multiple testing. P values < 0.05 were considered significant.

Regarding the N-glycan data, log-transformed glycan values were tested for associations using linear models. For all the analyses, age was included in the model as covariate. Benjamini–Hochberg correction for multiple testing was used with a significance threshold of 0.05.

The diagnostic potential of investigated parameters was assessed using receiver operating characteristic (ROC) curves of individual and combined parameters. The optimal cut-off was determined using Youden indexes. The impact of different combinations of parameters on the diagnostic potential of cfDNA in body liquids was assessed by performing logistic regression. All analyses were performed using R version 4.3.2.

## Results

### Clinicopathological data

The clinicopathological characteristics of NSE patients and healthy controls are shown in Table 1.

**Table 1:** Clinicopathological characteristics. NSE, nonseminomatous testicular germ cell tumor; EC, embryonal carcinoma; TE, teratoma; MTGCT, mixed testicular germ cell tumor; STM + , serum tumor marker positive patients; STM-, serum tumor marker negative patients

**Healthy controls**		**114**	
Age—median (range)		30 (18–44)	
**NSE patients**		**41**	
Age—median (range)		27 (20–46)	
		STM +	STM−
EC		5	5
TE		1	2
MTGCT		21	7
Clinical stage	I	36	
	II	2	
	III	3	
Pathological stage	T1	24	
	T2	13	
	T3	4	

### cfDNA parameters

Blood plasma.

### cfDNA amounts and CFI

**Fig. 1:**
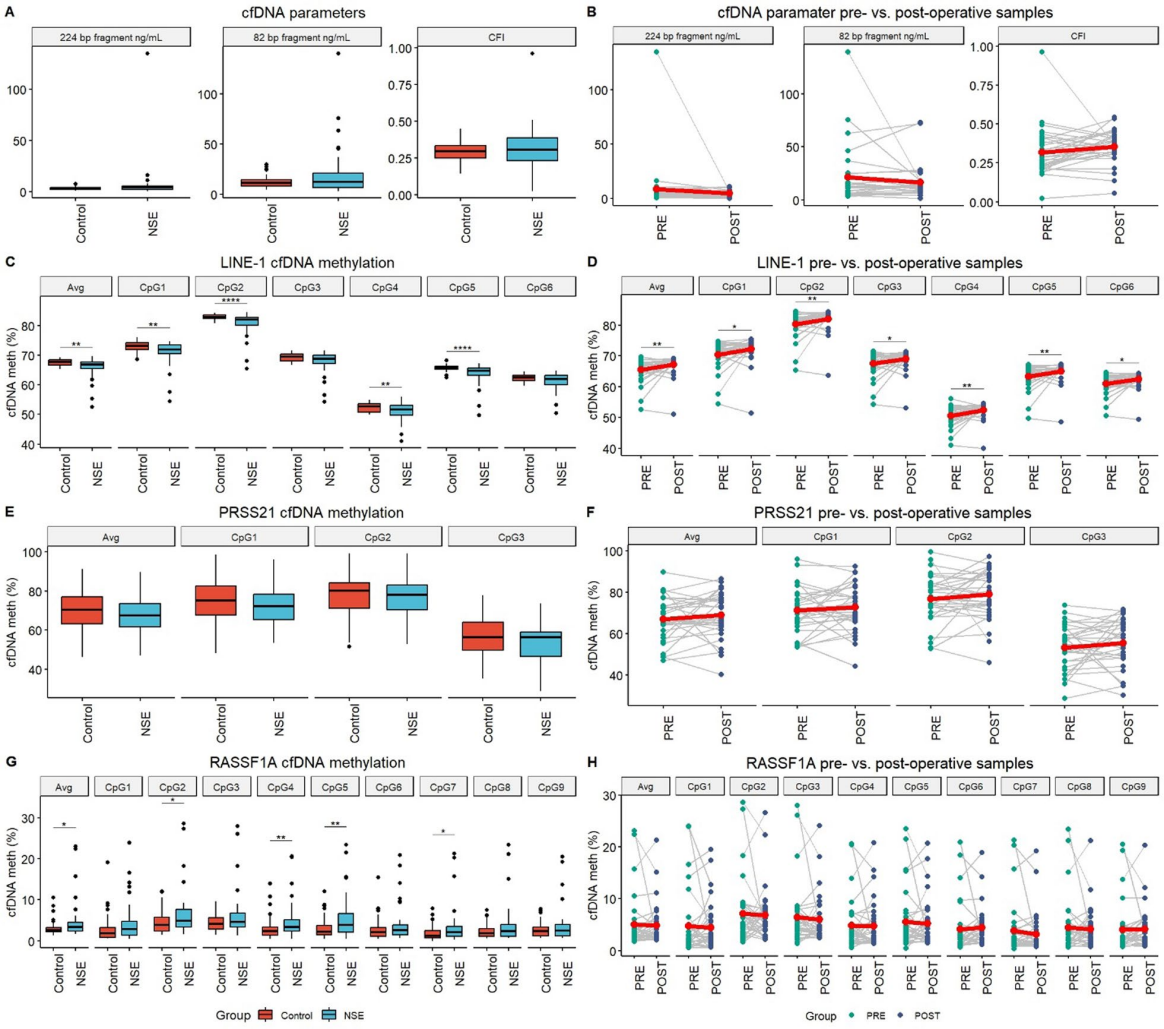
Investigated cfDNA parameters in blood plasma of NSE patients and healthy controls. **A** cfDNA fragment amounts and CFI. **B** Comparison of cfDNA fragment amounts and CFI between pre- and post-operative NSE patients. **C** *LINE-1* cfDNA methylation levels. **D** *LINE-1* cfDNA methylation levels between pre- and post-operative NSE patients. **E** *PRSS21* cfDNA methylation levels. **F** *PRSS21* cfDNA methylation levels between pre- and post-operative NSE patients. **G** *RASSF1A* cfDNA methylation levels. **H** *RASSF1A* cfDNA methylation levels between pre- and post-operative NSE patients. Statistically significant differences are indicated as *—p < 0.05; **—p < 0.01; ***—p < 0.001 and ****—p < 0.0001. Lineplots include a red dot representing the average of the group. Boxplots depict the median and IQR. *NSE* nonseminomatous testicular germ cell tumor patients, *PRE* preoperative patients samples, *POST* postoperative patients samples

No statistically significant difference in the amount of short or long cfDNA fragments or in CFI between NSE patients and healthy controls was detected in blood plasma (Fig. 1A). No statistically significant difference was detected either between preoperative or postoperative samples (Fig. 1B).

### cfDNA methylation analysis

Statistically significant hypomethylation of *LINE-1* was detected in blood plasma of NSE patients compared to healthy controls in CpG’s 1, 2, 4, 5 and the average of the region (Fig. 1C). Statistically significant hypermethylation was detected in postoperative NSE patient’s samples compared to preoperative samples across all investigated CpG’s. Postoperative patients’ samples *LINE-1* cfDNA methylation levels showed no statistically significant difference from healthy controls (Fig. 1D).

No statistically significant difference in *PRSS21* cfDNA methylation levels was detected in blood plasma between NSE patients and healthy controls (Fig. 1E). No statistically significant difference in *PRSS21* cfDNA methylation between preoperative and postoperative NSE patients’ samples was detected (Fig. 1F).

Statistically significant hypermethylation of *RASSF1A* cfDNA in NSE patients’ blood plasma was detected, in CpG’s 2, 4, 5, 7 and the average of the region (Fig. 1G). No statistically significant difference in cfDNA methylation was detected between preoperative and postoperative NSE patients’ samples (Fig. 1H).

### Blood plasma protein N-glycome

**Fig. 2:**
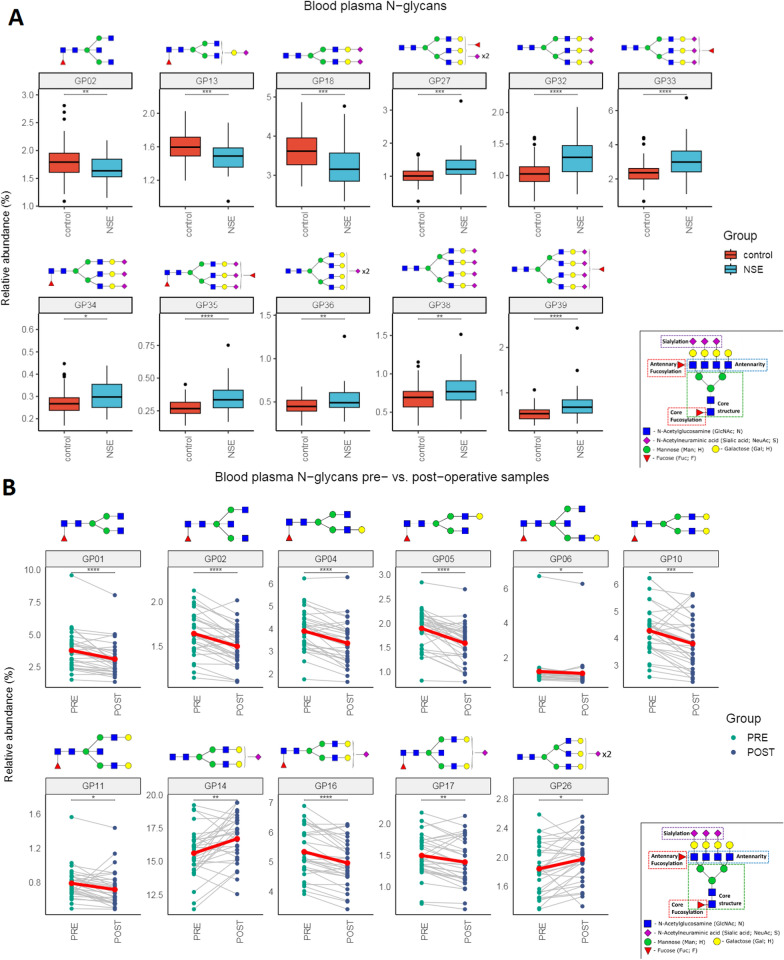
N-glycan profile in blood plasma. **A** changes of blood plasma protein N-glycosylation between NSE patients and healthy controls, shown as boxplots. **B** Longitudinal changes of blood plasma protein N-glycosylation between preoperative and postoperative samples, shown as lineplots. Only statistically significant glycan peaks are shown. Statistically significant differences are indicated as *—p < 0.05; **—p < 0.01; ***—p < 0.001 and ****—p < 0.0001. Lineplots include a red dot representing the average of the group. Boxplots depict the median and IQR. *NSE* nonseminomatous testicular germ cell tumor patients, *PRE* preoperative patients samples, *POST* postoperative patients samples

Blood plasma protein N-glycosylation chromatograms were separated into 39 N-glycan relatively quantified chromatographic peaks. Statistically significant changes in N-glycosylation profile of blood plasma proteins between NSE patients and healthy controls were detected in eleven glycan peaks. Namely, the proportions of glycan structures represented as GP02, GP13 and GP18 were decreased in NSE patients, while GP27, GP32, GP33, GP34, GP35, GP36, GP38 and GP39 were decreased in healthy controls. Significant associations are shown in (Fig. 2A). Regarding the longitudinal samples, once again significant changes of N-glycosylation were observed. Significant longitudinal changes between preoperative and postoperative N-glycan blood plasma protein profiles are presented in (Fig. 2B). GP01, GP02, GP04, GP05, GP06, GP10, GP11, GP16 and GP17 decreased in postoperative samples, while proportions of GP14 and GP26 showed an increase in relative abundance after surgery.

### Seminal plasma

#### cfDNA amounts and CFI

**Fig. 3:**
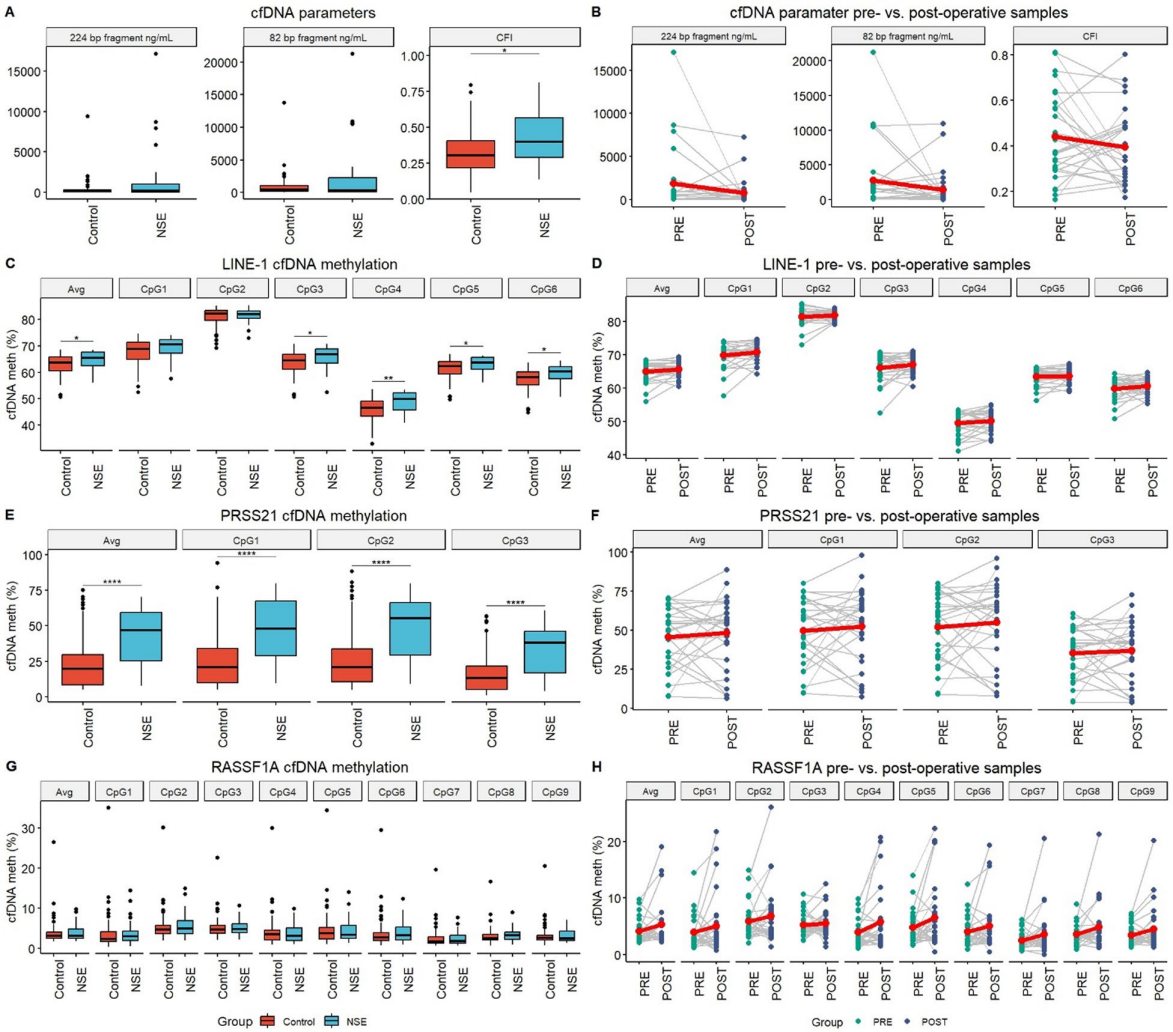
Investigated cfDNA parameters in seminal plasma of NSE patients and healthy controls. **A** cfDNA fragment amounts and CFI. **B** Comparison of cfDNA fragment amounts and CFI between pre- and post-operative NSE patients. **C** *LINE-1* cfDNA methylation levels. **D** *LINE-1* cfDNA methylation levels between pre- and post-operative NSE patients. **E** *PRSS21* cfDNA methylation levels. **F** *PRSS21* cfDNA methylation levels between pre- and post-operative NSE patients. **G** *RASSF1A* cfDNA methylation levels. **H** *RASSF1A* cfDNA methylation levels between pre- and post-operative NSE patients. Statistically significant differences are indicated as *—p < 0.05; **—p < 0.01; ***—p < 0.001 and ****—p < 0.0001. Lineplots include a red dot representing the average of the group. Boxplots depict the median and IQR. *NSE* nonseminomatous testicular germ cell tumor patients, *PRE* preoperative patients samples, *POST* postoperative patients samples

No statistically significant difference in the amounts of short or long cfDNA fragments has been detected in seminal plasma of NSE patients and healthy controls (Fig. 3A). A statistically significant increase in CFI in NSE patients was detected compared to healthy controls. No statistically significant difference between preoperative and postoperative NSE patients’ samples was detected (Fig. 3B).

#### cfDNA methylation analysis

Statistically significant hypermethylation of *LINE-1* cfDNA was detected in seminal plasma of NSE patients, in CpG’s 3, 4, 5, 6 and the average of the region (Fig. 3C). No statistically significant difference in *LINE-1* cfDNA methylation were detected between preoperative and postoperative NSE patients’ samples (Fig. 3D).

Statistically significant hypermethylation of *PRSS21* cfDNA in NSE patients’ seminal plasma was detected across all investigated CpG’s and the average of the region (Fig. 3E). No statistically significant difference was detected between preoperative and postoperative NSE patients’ samples (Fig. 3F).

No statistically significant difference in cfDNA methylation levels of *RASSF1A* in seminal plasma has been detected between NSE patients and healthy controls (Fig. 3G). No statistically significant difference was detected between preoperative and postoperative NSE patients’ samples (Fig. 3H).

### Seminal plasma protein N-glycome

Seminal plasma protein N-glycosylation chromatograms were separated into 31 N-glycan relatively quantified chromatographic peaks. No statistically significant differences of seminal plasma protein N-glycome between NSE patients and healthy controls were detected (Supplementary Fig. 1). No statistically significant difference was detected between preoperative and postoperative NSE patients’ samples (Supplementary Fig. 2).

### Diagnostic potential

To evaluate the diagnostic potential of biomarkers, logistic regression analysis was conducted on parameters that demonstrated statistical significance in both blood and seminal plasma and (Supplementary Table 2).

**Fig. 4:**
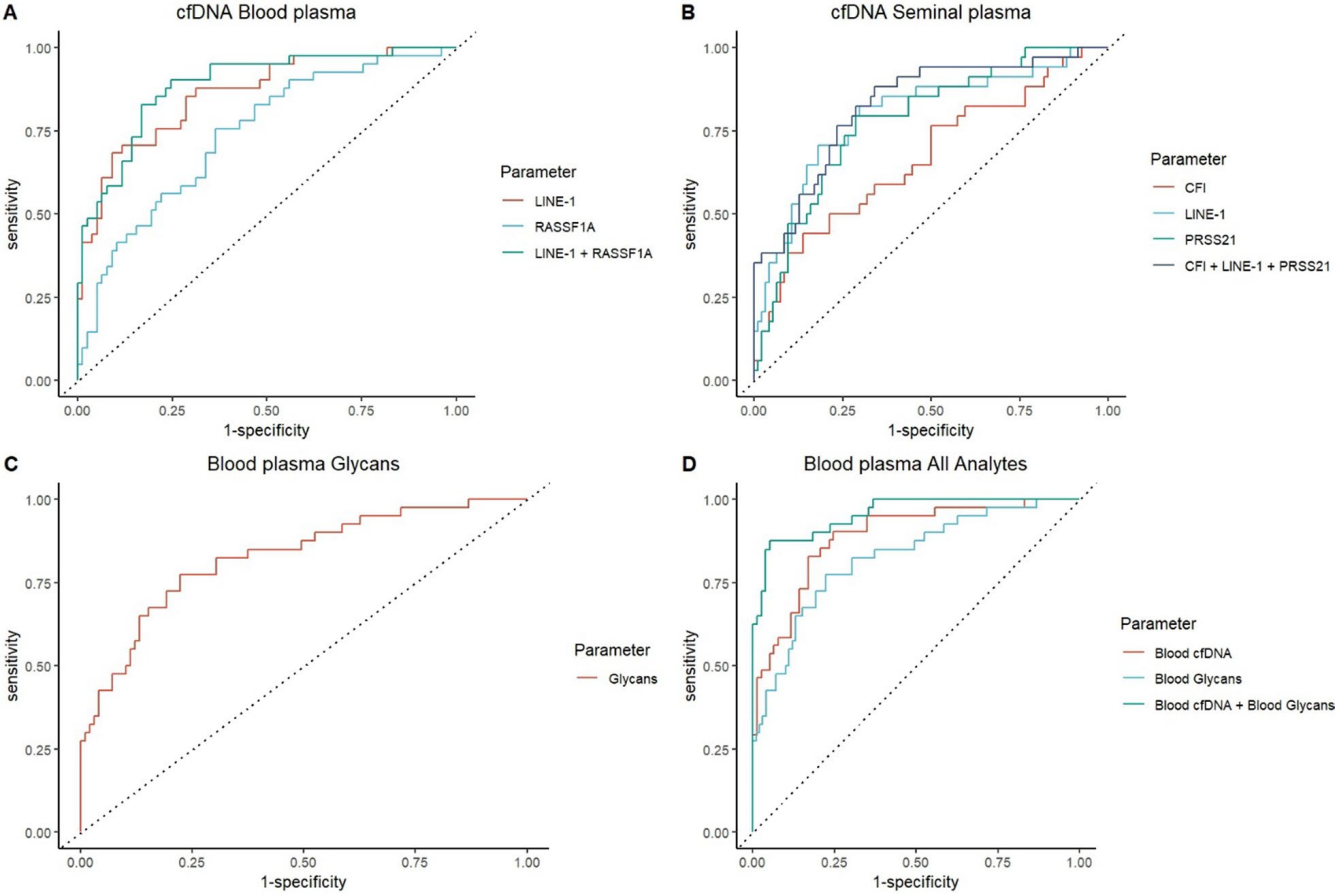
ROC curves assessing the diagnostic potential of analytes in discriminating NSE patients from healthy controls. **A** cfDNA parameters in blood plasma. **B** cfDNA parameters in seminal plasma. **C** N-glycan profile in blood plasma. **D** Combined cfDNA and N-glycan parameters in blood plasma

In blood plasma *LINE-1* cfDNA methylation has an AUC of 0.86 and a sensitivity/specificity of 68%/90% in discriminating NSE patients from healthy controls (Fig. 4A). *RASSF1A* cfDNA methylation has an AUC of 0.74 and a sensitivity/specificity of 76% / 64% in discriminating NSE patients from healthy controls. The two combined have an AUC of 0.89 and a sensitivity/specificity of 83%/83% in discriminating NSE patients from healthy controls. Using blood plasma cfDNA we have detected 79% of STM negative patients and have detected all teratoma patients present in this study.

In seminal plasma CFI has shown an AUC of 0.67 and a sensitivity/specificity of 44% / 86% in discriminating NSE patients from healthy controls (Fig. 4B). *LINE1* cfDNA methylation has an AUC of 0.80 and a sensitivity/specificity of 82% / 70% in discriminating NSE patients from healthy controls. *PRSS21* cfDNA methylation has an AUC of 0.78 and a sensitivity/specificity of 79% / 71% in discriminating NSE patients from healthy controls. The three combined have an AUC of 0.83 and a sensitivity/specificity of 88% / 65% in discriminating NSE patients from healthy controls. Using seminal plasma cfDNA parameters we have detected 85% of STM negative patients and two out of the three teratoma patients present in this study.

The blood plasma N-glycan profile of NSE patients has shown an AUC of 0.83 and a sensitivity/specificity of 78% / 78% in discriminating NSE patients from healthy controls (Fig. 4C). Using blood plasma’s N-glycan profile we have managed to detect 69% of STM negative patients and have detected 50% of teratoma patients. 

Finally, the combination of cfDNA and N-glycan profile in blood plasma has shown an AUC of 0.96 and a sensitivity/specificity of 88% / 95% in discriminating NSE patients from healthy controls (Fig. 4D). Using the combined cfDNA parameters and N-glycan profile in blood plasma we have detected 85% of STM negative patients and all teratoma patients present in this study.

## Discussion

Our study provides novel insights into the diagnostics of NSE by highlighting the limitations of relying solely on single-analyte liquid biopsy analyses for complex diseases like TGCTs [33, 40, 41]. Using a multi-analyte approach, combining cfDNA parameters and N-glycan profiles in both blood and seminal plasma, addresses these challenges. This included the first-time investigation of cfDNA methylation patterns for *LINE-1*, and *PRSS21* and N-glycosylation patterns as well as validation of *RASSF1A* cfDNA methylation in blood plasma, alongside CFI assessments in these body liquids. This marks a significant departure from the prevailing focus on microRNA, especially miR-371a-3p, which despite its high sensitivity and specificity and its probable clinical adoption, is unable to detect teratomas [11, 35, 42].

### TGCT patients

#### cfDNA profile

We investigated the cfDNA profile of NSE patients by analyzing cfDNA amounts and fragmentation patterns. Unlike previous studies which investigated cfDNA amounts in blood samples we did not observe an increase in overall cfDNA amounts in either blood or seminal plasma. Similarly contradictory reports on CFI were reported, with one study detecting an increase and one a decrease in CFI in blood samples, while we observed no change in blood plasma CFI levels [43, 44]. However, we detected an increase in the CFI of seminal plasma of NSE patients, demonstrating an AUC of 0.67 with 44% sensitivity and 86% specificity. This increase likely reflects the increase in di-nucleosomal cfDNA previously observed in blood plasma of TGCT patients [44]. Given the variability in CFI responses across cancer types [45, 46], our detection of increased CFI asks for further investigation into longer cfDNA fragments (> 360 bp) to provide deeper insights in NSE patients. 

#### cfDNA methylation levels

We examined the methylation patterns of *LINE-1, PRSS21* and *RASSF1A* in cfDNA from both blood and seminal plasma of NSE patients. Hypomethylation of *LINE-1* cfDNA in NSE patients’ blood plasma showed significant diagnostic potential (AUC of 0.86, 68% sensitivity, 90% specificity), in contrast with previous studies on whole blood DNA of TGCT patients which may have been obscured by non-cancerous genomic DNA [47]. In seminal plasma *LINE-1* cfDNA hypermethylation was detected (AUC of 0.80, 82% sensitivity, 70% specificity). The opposite directions of cfDNA methylation between these body liquids can be explained by taking into consideration its cellular origin. Blood plasma cfDNA has a relatively low genomic DNA background and so in NSE patients a significant amount of blood plasma cfDNA is tumor derived and therefore hypomethylated. Seminal cfDNA in contrast is much more abundant and therefore has a higher background of genomic DNA (mostly from sperm cells, testicular tissue, monocytes, granulocytes and prostate tissue) [48]. These tissues are known to undergo *LINE-1* hypermethylation due to oxidative stress [49], which is significantly increased in patients with testicular cancer [50] suggesting we are detecting changes in the surrounding tissue caused by the tumor. The overall changes in *LINE-1* cfDNA methylation levels suggests its efficacy as a biomarker across different bodily fluids, confirming it as a hallmark of malignant transformation [18, 51]. *PRSS21* cfDNA methylation did not show significant changes in blood plasma between NSE patients and controls, likely due to the blending of highly methylated *PRSS21* cfDNA levels in healthy plasma with highly methylated tumor *PRSS21 cfDNA* [17, 52]. Hypermethylation of *PRSS21* in seminal plasma (AUC of 0.78, 79% sensitivity, 71% specificity) has been shown to be a specific diagnostic marker underscoring the diagnostic relevance of seminal fluid in TGCT research. *RASSF1A* cfDNA hypermethylation in blood plasma of NSE patients (AUC of 0.74, 76% sensitivity, 64% specificity), validates its diagnostic value [26, 53]. The detected hypermethylation is on the same CpG’s as a previous study investigating whole blood *RASSF1A* methylation, verifying the epigenetic significance of the investigated region [54]. In seminal plasma we have not detected significant changes in *RASSF1A* cfDNA methylation, reinforcing the specificity of cfDNA methylation patterns to the body fluid type.

### Plasma proteins N-glycosylation

Our study presents an exclusive analysis of blood plasma protein N-glycosylation in NSE. We identified eleven distinct N-glycan alterations in the blood plasma of NSE patients compared to healthy controls, primarily in complex multiantennary glycans. The most notable changes were observed in GP18 and GP34 structures (corresponding to structures A2G2S2 and FA3G3S3 respectively), consistent with previous research [34], and both could potentially originate from bHCG, a recognized TGCT marker [33, 55–58]. This connection is especially interesting in the FA3G3S3 structure, known to be elevated in tumor cell-derived bHCG (particularly in choriocarcinoma) highlighting its role in tumor progression and malignancy development [58–61]. The detected N-glycan changes may have their origin in alpha-1-acid glycoprotein, which exhibits altered glycosylation in various malignancies [62–64]. Despite the abundant presence of N-linked glycoproteins in seminal plasma, no significant N-glycan differences were detected between NSE patients and healthy controls. This disconnect emphasizes the complexity of TGCT biomarker research and highlights the need for further exploration of glycosylation patterns in different body fluids, potentially shedding light on a causative role of N-glycosylation in malignancy development.

### Assessment of curative procedure

We assessed the utility of liquid biopsy analytes for evaluating the success of radical orchidectomy in TGCT. Post-operative blood plasma samples showed that *LINE-1* cfDNA methylation levels returned to those of healthy controls, suggesting it as a novel biomarker for post-operative recovery. Additionally, in two out of three clinical stage III patients this recovery was not observed. This discovery could potentially allow for more nuanced therapeutic decisions for stage I NSE patients, particularly to avoid overtreatment in TGCT management [14]. Additionally, alterations in eleven glycan peaks following surgery were detected, however without a definitive return to levels observed in healthy controls. This suggests the potential of blood plasma N-glycans as biomarkers for post-operative outcome, warranting further longitudinal studies to illuminate their role in the recovery process.

### Body liquid biomarker potential

**Fig. 5:**
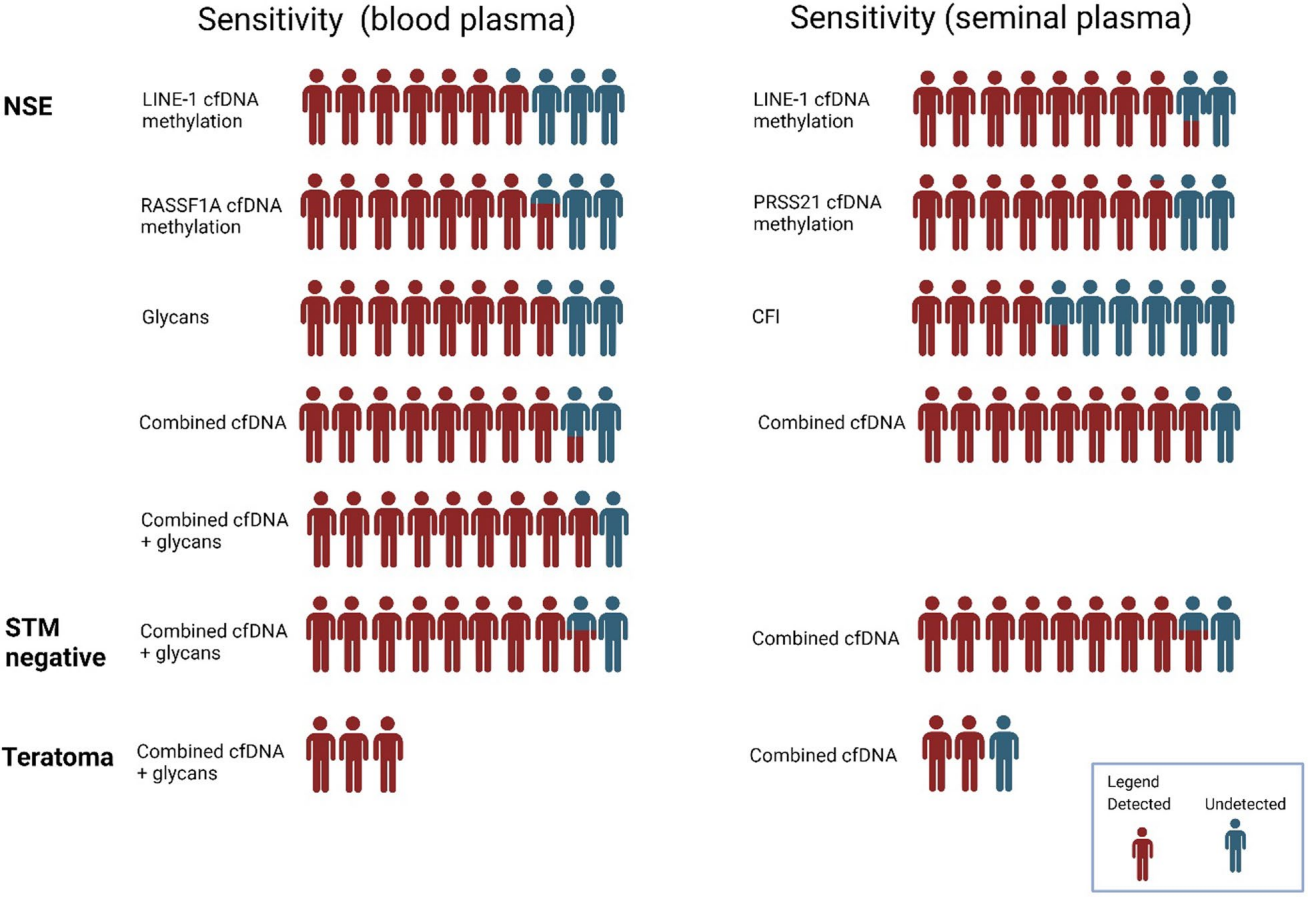
Sensitivity of the investigated parameters for diagnostics of NSE patients in blood plasma and seminal plasma. Human icons are used to visually represent the sensitivity of different biomarkers across patient subgroups. The proportion of red to blue colour illustrates the sensitivity (detection rate percentage) for each approach. Each row represents a different biomarker or combination of biomarkers and they are grouped into three blocks: NSE, STM-negative patients, and teratoma patients

Liquid biopsy research has mainly focused on blood which has shown limitations especially in detection of cfDNA from encapsulated organs such as the prostate or testis [11, 36, 65]. Different body liquids have been analyzed to overcome this limitation and ejaculate has shown potential in prostate cancer and infertility research [11, 66–70]. In this research we have determined diagnostic potentials of analytes in body liquids using logistic regression and they have shown in blood plasma cfDNA an AUC of 0.89, in seminal plasma cfDNA an AUC of 0.83 and an AUC of 0.83 for the N-glycan profile in blood plasma. The combined analysis of cfDNA and N-glycan profiles in blood plasma achieved an AUC of 0.96, improving the detection reliability for STM-negative NSE patients and specifically identifying all pure teratoma cases within our cohort (Fig. 5). While this confirms the potential seminal plasma has in male health diagnostics, we have overall found blood plasma the more applicable and easily implementable liquid biopsy source for TGCT diagnostics, aligning with ESMO guidelines [71].

The pressing clinical need in TGCT diagnostics is the detection of TE and STM-negative patients [11, 72]. In comparison to miR-371a-3p, the most perspective TGCT biomarker [11, 73], our suggested multianalyte approach is comparable in diagnostic potential. However, while miR-371a-3p’s efficacy has been investigated across a number of studies, is more easily adoptable in a clinical setting, is very cost effective and is in ongoing clinical trials [73] there remains an unresolved issue, namely the inability of miR-371a-3p to detect TE patients [11, 35]. While attempts have been made to bridge this gap using miR-375-3p and miR-375-5p [74] the initial findings could not be verified in further studies [75–77]. More importantly, in a study by Lafin et al. small RNA sequencing found no candidate microRNA in blood serum for TE detection [75]. However, in a study performed by Lobo et al. they supplemented miR-371a-3p detection with *RASSF1A* cfDNA methylation and managed to detect patients with TE [26]. Our approach confirms that miR-371a-3p supplementation for TE detection should be using cfDNA methylation, or a multianalyte test for potentially even greater accuracy.

### Study limitations and future research directions

Despite the promising results, our study has several limitations. Firstly, the number of recruited NSE patients is relatively small. Studies on larger cohorts or more studies combined into a metanalysis are required to validate our findings and ensure their generalizability. Secondly, the longitudinal study is focused within a short timeframe. Long-term follow-up studies are necessary to evaluate the stability and prognostic value of cfDNA and N-glycan biomarkers over time. Thirdly, while the study shows the potential of seminal plasma as a diagnostic fluid, more extensive research is required to understand the variability of seminal plasma analysis.

Addressing these limitations will enhance the robustness of the diagnostic methods proposed and contribute to the development of more reliable and widely applicable TGCT diagnostic tools.

## Conclusion

Our multi-analyte approach, combining cfDNA parameters and N-glycan profiling in blood plasma accurately detects NSE patients with an AUC of 0.96. This approach is particularly effective in identifying STM-negative cases and pure-form teratomas, addressing a critical unmet need in TGCT diagnostics. Additionally, we have demonstrated the utility of seminal plasma as a valuable diagnostic fluid for patients with TGCT. Finally, *LINE-1* cfDNA methylation presents itself as a promising multi body fluid biomarker for NSE detection and a novel marker for monitoring post-operative success. These findings highlight the potential of cfDNA parameters and N-glycan profiles in improving TGCT diagnostics, offering a more comprehensive and precise approach to patient detection and treatment guidance. Future studies should focus on validating these biomarkers in larger, multicenter cohorts and exploring their integration into clinical workflows to improve patient outcomes and reduce overtreatment.

## Supplementary Information

Supplementary file 1.

Supplementary file 2.

Supplementary file 3.

Supplementary file 4.

## Data Availability

All data generated or analyzed during this study are available from the corresponding author on reasonable request.

## Abbreviations

AFP: α-Fetoprotein
AUC: Area under curve
bHCG: β-Human chorionic gonadotropin
cfDNA: Cell-free DNA
CFI: Cell-free DNA fragmentation index
CH: Choriocarcinoma
EC: Embryonal carcinoma
LDH: Lactate dehydrogenase
MTGCT: Mixed testicular germ cell tumor
NSE: Nonseminomatous testicular germ cell tumor
ROC: Receiver operating characteristic
SE: Seminomatous testicular germ cell tumor
STM: Serum tumor marker
TE: Teratoma
TGCT: Testicular germ cell tumors
YST: Yolk sac tumor
