# Supplementary material for: A multi-analyte liquid biopsy approach for nonseminomatous testicular germ cell tumors: combining cfDNA and N-glycan analysis in blood and seminal plasma
Source: Cancer Cell Int. 2025 Jul 11;25:257. doi: 10.1186/s12935-025-03887-8 (PMC12247211; doi:10.1186/s12935-025-03887-8)
Supplement: Supplementary file 1 [file 12935_2025_3887_MOESM1_ESM.pdf]

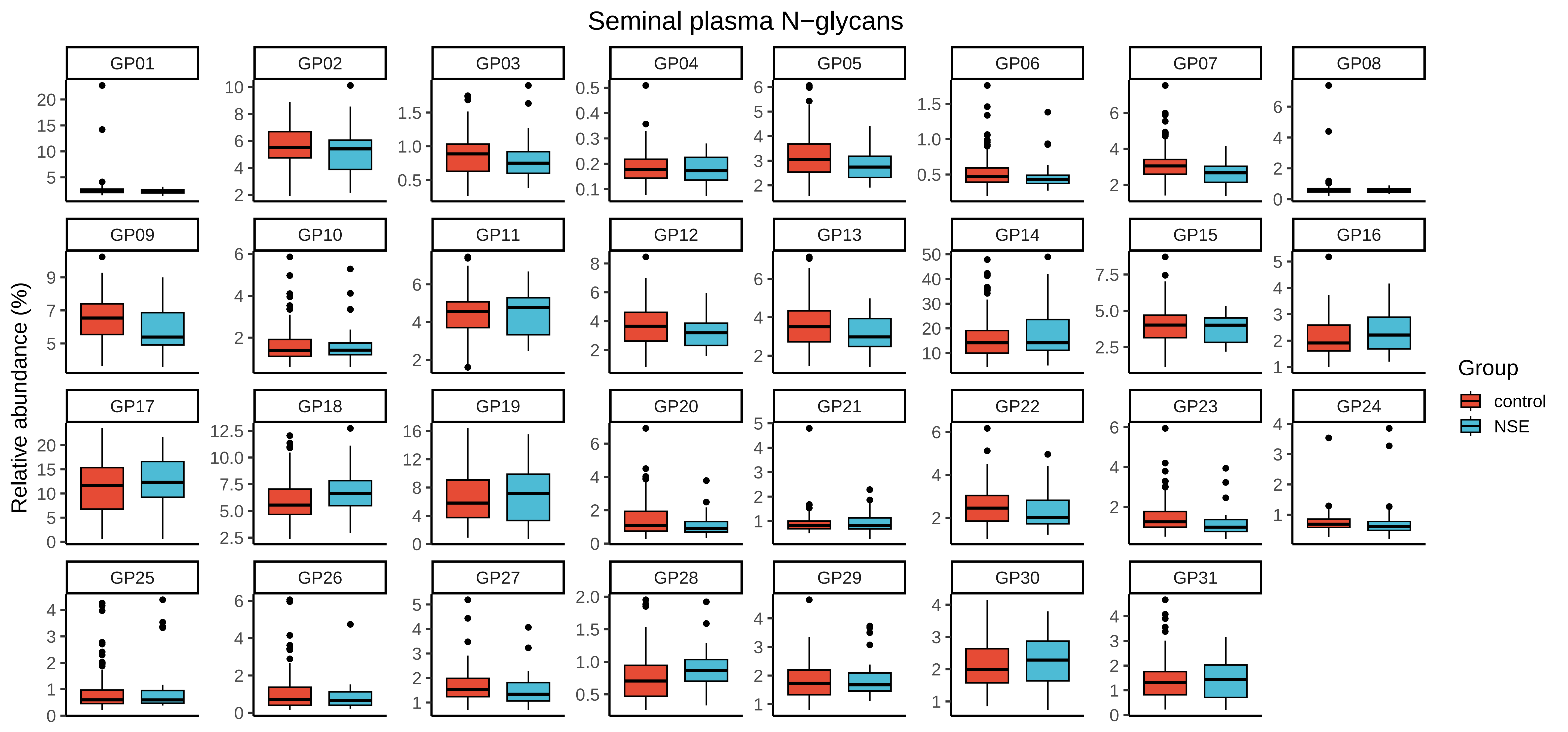
Seminal plasma N−glycans
GP01
GP02
GP03
GP04
GP05
GP06
GP07
GP08
GP09
GP10
GP11
GP12
GP13
GP14
GP15
GP16
GP17
GP18
GP19
GP20
GP21
GP22
GP23
GP24
GP25
GP26
GP27
GP28
GP29
GP30
GP31
Relative abundance (%)
Group
control
NSE

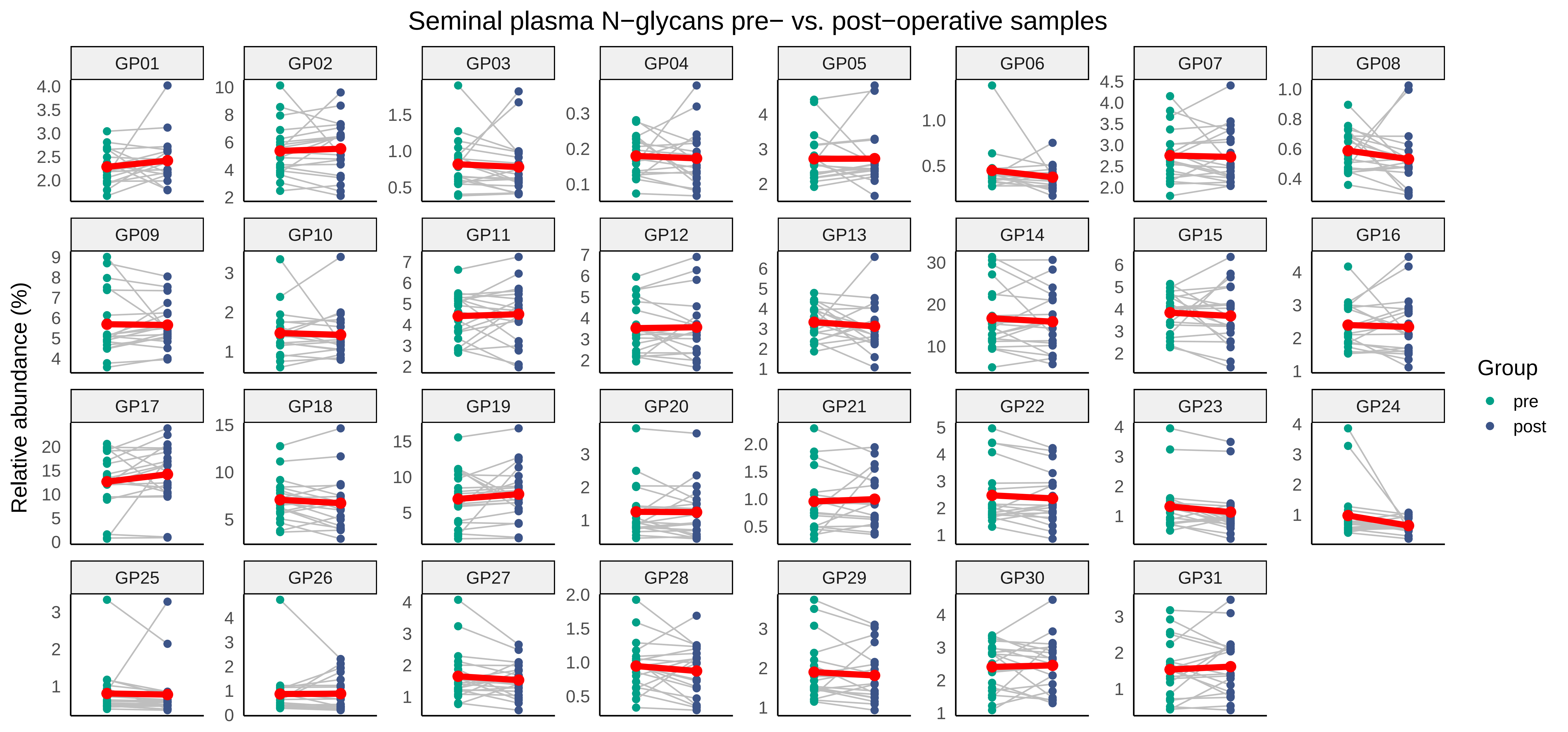
Seminal plasma N−glycans pre− vs. post−operative samples
Relative abundance (%)
GP01
GP02
GP03
GP04
GP05
GP06
GP07
GP08
GP09
GP10
GP11
GP12
GP13
GP14
GP15
GP16
GP17
GP18
GP19
GP20
GP21
GP22
GP23
GP24
GP25
GP26
GP27
GP28
GP29
GP30
GP31
Group
pre
post

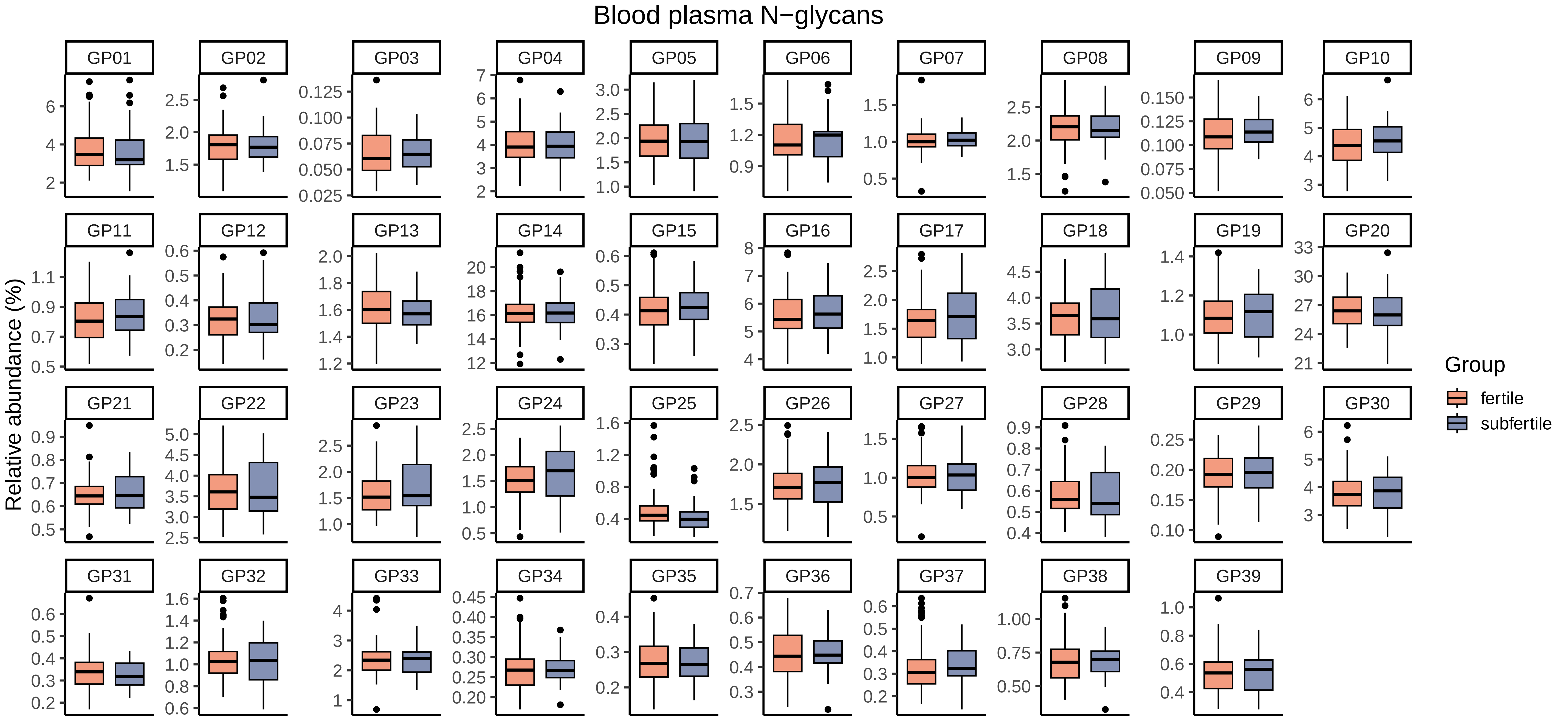

Blood plasma N−glycans
Relative abundance (%)
GP01
GP02
GP03
GP04
GP05
GP06
GP07
GP08
GP09
GP10
GP11
GP12
GP13
GP14
GP15
GP16
GP17
GP18
GP19
GP20
GP21
GP22
GP23
GP24
GP25
GP26
GP27
GP28
GP29
GP30
GP31
GP32
GP33
GP34
GP35
GP36
GP37
GP38
GP39
Group
fertile
subfertile

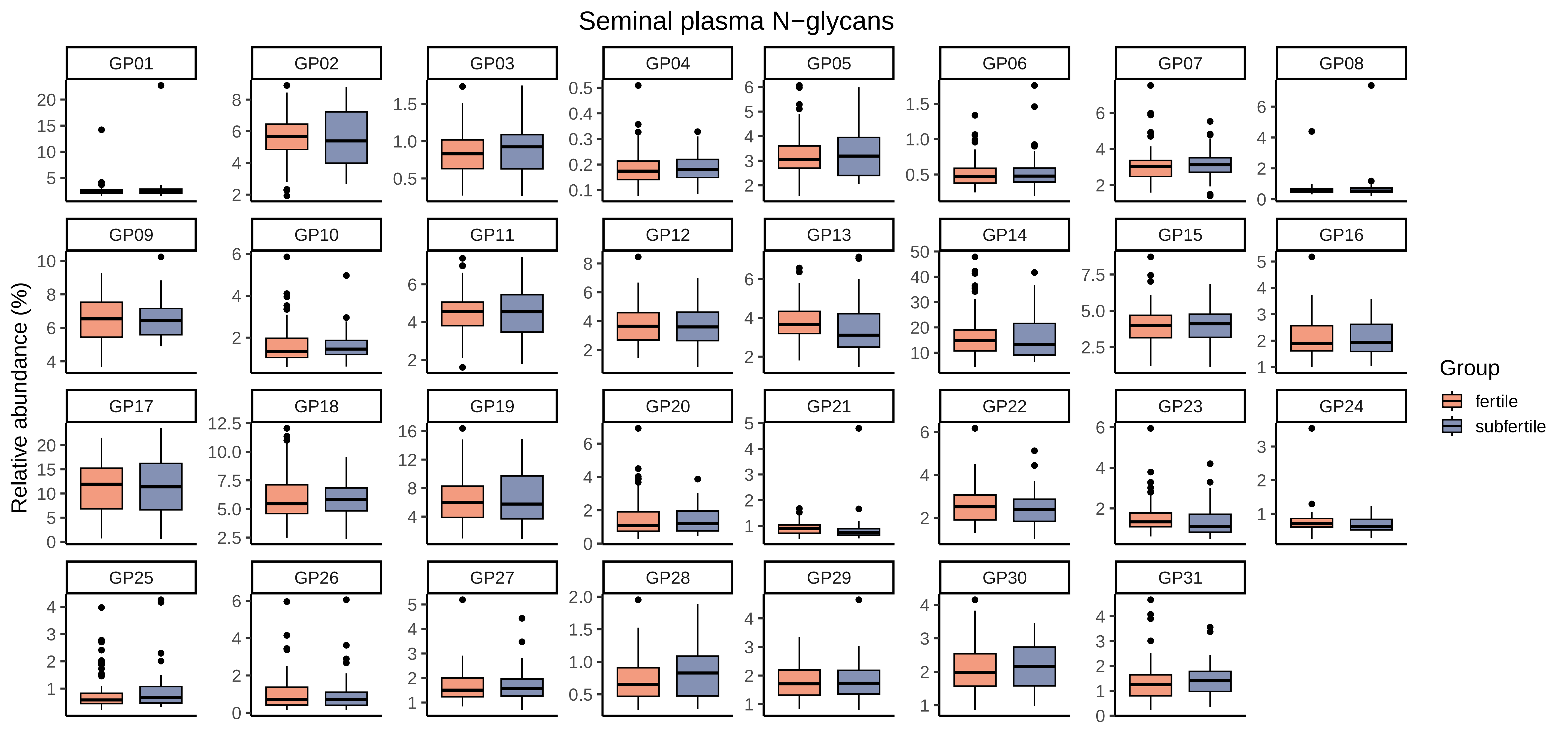

Seminal plasma N−glycans
Relative abundance (%)
GP01
GP02
GP03
GP04
GP05
GP06
GP07
GP08
GP09
GP10
GP11
GP12
GP13
GP14
GP15
GP16
GP17
GP18
GP19
GP20
GP21
GP22
GP23
GP24
GP25
GP26
GP27
GP28
GP29
GP30
GP31
Group
fertile
subfertile

Supplementary Table 1. Primer and PCR information

Gene	Primer	Sequence	bp	temp	No. Cycles
PRSS21	F	5'-GTAGATAAGAAAGGGATTGTG-3'	128	56°C	50
	R	5'-BIOT- CCCAAAAACTATTTAAAACCTCTCAT-3'			
	SEQ	5'-GGATTGTGGGGTTTA-3'			
	SEQ ANA	5'-TYGGGTTTGGGTTATATTAAGAAGTGTGGTTGAAGATTYGTTTTTAGGGGTTGAAAGTTAGGGYGTTGTTAGGTATGAGAGGTTTTAAA-3'			
RASSF1A	F	5'-AGTTTGGATTTTGGGGGAGG-3'	136	65°C	50
	R	5'-BIOT-CAACTCAATAAACTCAAACTCCCC-3'			
	SEQ	5'-GGGTTAGTTTTGTGGTTT-3'			
	SEQ ANA	5'-YGTTYGGTTYGYGTTTGTTAGYGTTTAAAGTTAGYGAAGTAYGGGTTTAATYGGGTTATGTYG-3'			
LINE-1	F	5'-BIOT-TAGGGAGTGTTAGATAGTGG-3'	120	58°C	50
	R	5'-AACTCCCTAACCCCTTAC-3'			
	SEQ	5’-CAAATAAAACAATACCTC-3’			
	SEQ ANA	5'-RCCCTACTTCRACTCRCRCACRATACRCACACACACTAACCTAC-3'			
