# Supplementary material for: A multi-analyte liquid biopsy approach for nonseminomatous testicular germ cell tumors: combining cfDNA and N-glycan analysis in blood and seminal plasma
Source: Cancer Cell Int. 2025 Jul 11;25:257. doi: 10.1186/s12935-025-03887-8 (PMC12247211; doi:10.1186/s12935-025-03887-8)
Supplement: Supplementary file 2 [file 12935_2025_3887_MOESM2_ESM.pdf]

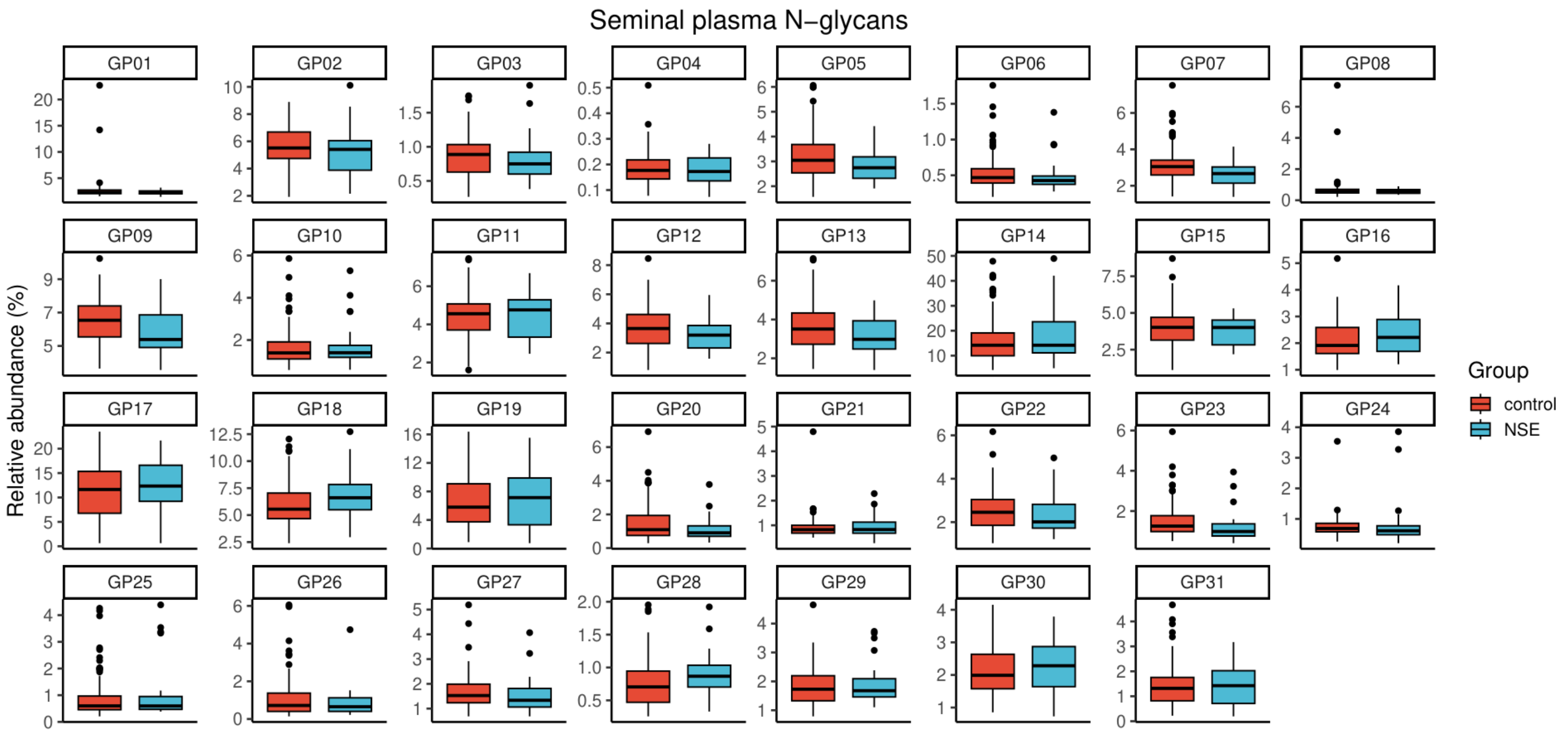

Supplementary Figure 1 – Changes of seminal plasma protein N-glycosylation between NSE patients and healthy controls, shown as boxplots depicting the median and IQR. Statistically significant differences are indicated as * - p < 0.05; ** - p < 0.01; *** - p < 0.001 and **** - p < 0.0001. NSE - nonseminomatous testicular germ cell tumor patients.

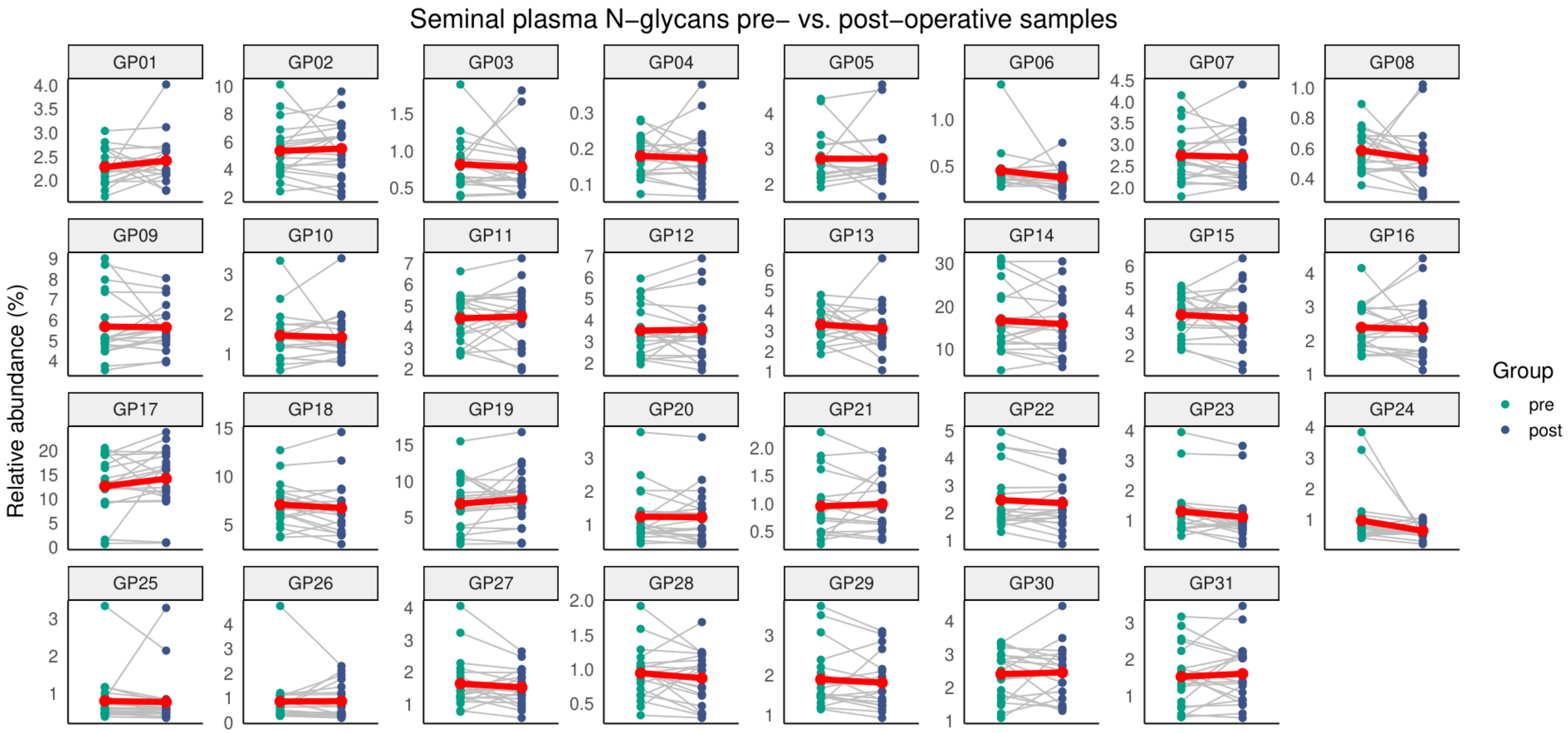

Supplementary Figure 2 – Longitudinal changes of blood plasma protein N-glycosylation between preoperative and postoperative samples, shown as lineplots. Statistically significant differences are indicated as * - p < 0.05; ** - p < 0.01; *** - p < 0.001 and **** - p < 0.0001. Lineplots include a red dot representing the average of the group. PRE – preoperative patients samples, POST – postoperative patients samples.
